# Feasibility of Home-Based Early Infant Hybrid Therapy in Children with Unilateral Cerebral Palsy

**DOI:** 10.3390/jcm13226725

**Published:** 2024-11-08

**Authors:** Rocío Palomo-Carrión, Helena Romay-Barrero, Elena Pinero-Pinto, Rita-Pilar Romero-Galisteo, María Coello-Villalón, Asunción Ferri-Morales, Purificación López-Muñoz, Cristina Lirio-Romero

**Affiliations:** 1 Faculty of Physiotherapy and Nursing of Toledo, University of Castilla-La Mancha, Avenida Carlos III, s/n, 45071 Toledo, Spain; rocio.palomo@uclm.es (R.P.-C.); maria.coello@uclm.es (M.C.-V.); asuncion.ferri@uclm.es (A.F.-M.); purificacion.lopez@uclm.es (P.L.-M.); cristina.lirio@uclm.es (C.L.-R.); 2 ImproveLab, Research Group in Pediatric Physiotherapy and Neurology, 45071 Toledo, Spain; 3 Department of Physiotherapy, Faculty of Nursing, Physiotherapy and Podiatry, University of Seville, Avicena Street s/n, 41009 Seville, Spain; epinero@us.es; 4 Biomedicine Institute of Seville (IBIS), 41013 Seville, Spain; 5 Department of Physiotherapy, Faculty of Health Sciences, University of Málaga, C/Arquitecto Peñalosa, 29071 Málaga, Spain; rpromero@uma.es; 6 Biomedicine Institute of Málaga (IBIMA), 29590 Málaga, Spain

**Keywords:** early age, family, home, infantile hemiplegia, intensive therapy, upper extremity

## Abstract

**Background**: The first stage of childhood is characterized by great neuronal plasticity. In Unilateral Cerebral Palsy (UCP), it is essential to carry out early treatment, with family involvement. The aim of this study was to investigate the feasibility of Early Infant Hybrid Therapy (eI-Hybrid) applied at home with family involvement in children with UCP aged 9–18 months, and to assess its preliminary effectiveness on bimanual functional performance. **Methods**: A single group of 10 children (12.8 months, SD = 3.4) performed the eI-Hybrid therapy. The main outcome was measured with the mini Assisting Hand Assessment scale (mini-AHA), functional goals were measured with the Goal Attainment Scale (GAS), and satisfaction expectations on intensive therapy were also recorded. Three measures were performed (week 0, week 10, and month 6). A repeated-measures ANOVA test was performed on the mini-AHA in order to observe the statistically significant differences in pairwise comparison. **Results**: Ten children completed the study and the parents’ expectations were fulfilled, indicating high caregiver compliance and high adherence to the treatment. Clinically relevant changes were observed between pre- and post-intervention measurements in BFP (pre: 41.9 (SD: 7.7), post: 50.9 (SD: 6.0) and in the follow-up at 6 months (50.3 (SD:5.6); *p* < 0.001). Families reported a high satisfaction. **Conclusions**: infant hybrid treatment is feasible to be performed at home with the family’s involvement, obtaining improvements in the affected upper limb for early-age UCP.

## 1. Introduction

Cerebral palsy (CP) is the most common motor disability in childhood, characterized as a group of lifelong disorders affecting movement and posture, resulting in activity limitations [1]. Unilateral cerebral palsy (UCP) represents approximately 30% of all CP cases [2]. Children with UCP are specifically associated with sensorimotor deficits predominantly on one side of the body. These challenges typically manifest as difficulties with tasks involving grasping, releasing, and manipulating objects. Therefore, children with UCP experience the impaired function of one upper limb, which can hinder their ability to perform bimanual activities and negatively impact their overall development [3].

The remarkable plasticity of the young brain, especially within the first two years of life, underscores the importance of initiating therapeutic interventions at an early age [4]. Early training programs designed to enhance hand function are crucial in mitigating the risk of “developmental disregard”, where the affected limb is neglected by the child [5]. These programs should include age-specific interventions with therapy goals that are both task- and context-specific to maximize their effectiveness. Research indicates that certain motor impairments associated with CP can be partially reversed during this critical developmental window through early, intensive motor skill training [4].

Furthermore, studies emphasize the importance of actively involving parents and caregivers in the therapeutic process. Therapists should guide and support parents, helping them build their capacity and confidence, which in turn strengthens the parent–infant relationship and improves treatment adherence [6]. When parents feel well-supported, they are more likely to be actively involved in therapy, which can reduce their stress levels and enhance the effectiveness of the intervention. This is particularly important since early intervention programs are often home-based, relying on parents to deliver therapy [7].

Early therapeutic intervention carried out at home is critical during infancy, as it coincides with a period of rapid neural plasticity, offering a unique opportunity to influence motor outcomes positively [8]. Research has demonstrated that interventions within the first two years of life can significantly impact the motor and cognitive development of children with UCP [9]. Among these interventions, a recent review showed a small effect in the comparison of task-specific motor training with standard care for improving motor function, and a moderate effect for constraint-induced movement therapy (CIMT), compared with bimanual play or massage for improving the function of the more-affected hand [10]. Modified Constraint-Induced Movement Therapy (mCIMT) involves concurrent physical restraint of the less-affected hand and unilateral training of the more-affected hand and is typically less intensive than CIMT and flexible in terms of session length or frequency [11,12]. Bimanual Intensive Therapy (BIT) is a functional training method that uses intensive repetitive practice to increase both hands’ coordination with structured task practice through bimanual play and functional activities [13,14]. BIT and mCIMT have both shown promise in improving upper limb function in children older than 2 years [15,16,17]. Emerging evidence [18,19,20] suggests that combining these two approaches—creating an eI-Hybrid protocol—may offer enhanced therapeutic benefits by simultaneously promoting the use of the affected limb while also fostering the development of bimanual coordination [21,22].

The theoretical and practical benefits of the application of an early infant hybrid therapy (eI-Hybrid) approach in infants are widely demonstrated in the scientific literature, its use being recommended to improve upper limb function for children with UCP [15,19,23]. However, in young children, specifically in those aged 9–18 months, this remains largely unexplored. This age group presents unique challenges and opportunities, given their rapid developmental changes and the need for age-appropriate therapy protocols [24]. Understanding the feasibility of implementing such a hybrid intervention in this critical developmental window is essential for designing effective early intervention strategies.

The purpose of this study is to assess the bimanual functional performance increase in the affected upper limb and the feasibility of infant hybrid therapy applied at home with family involvement in children with UCP aged 9–18 months.

## 2. Materials and Methods

### 2.1. Study Design

The design selected was a single-group study, to test the feasibility of an early infant hybrid home protocol (eI-Hybrid). This protocol consisted of 50 h of intensive training (involving at least two sessions per week for a period of time) [25] divided into 25 h of bimanual intensive therapy (BIT) and 25 h of modified Constraint-Induced Movement Therapy (mCIMT). The parents were the main deliverers of the intervention. Before the study began, parents were provided with the written informed consent.

This study was registered in ClinicalTrials.gov with the identification number NCT06191588.

A single-group design was selected for this initial feasibility study due to its focus on evaluating the practicality and potential impact of the intervention within a natural home setting. Including a control group was considered but deemed premature, as the main objective was to explore whether parents could effectively implement the eI-Hybrid protocol and whether children would tolerate and respond to the therapy. Additionally, this design allowed for the refinement of the protocol before advancing to more complex, controlled trials.

To mitigate potential biases, several strategies were implemented. Firstly, standardized outcome measures [26,27,28,29,30] were used to reduce observer bias. Secondly, parents received detailed training to ensure consistency in administering the intervention. Thirdly, the data collection process was blinded to the maximum extent possible, with independent assessors evaluating the pre- and post-intervention outcomes. These measures aimed to enhance the reliability of the findings, despite the absence of a control group.

### 2.2. Participants

The participants were recruited from pediatric hospitals, specifically 12 de Octubre and La Paz in Madrid, and the University Hospital of Toledo, as well as from the association of unilateral cerebral palsy (HEMIWEB) in Spain, based on the established inclusion and exclusion criteria. The inclusion criteria were as follows: diagnosis of spastic UCP; being 9–18 months old; being able to understand and respond to basic and age-appropriate instructions (short, simple words with a clear order—take, drop, give me, move the toy, for example—used without generating possible doubts and with understanding for the young child); and the agreement from the parents to participate. Exclusion criteria included additional medical issues such as respiratory problems, which can affect a child’s ability to participate effectively in physical activities (e.g., asthma or chronic bronchitis), as well as intractable epilepsy and prior treatments such as medications, botulinum toxin injections, and surgeries. Additionally, a score of 4 points in the amount of use and initially used items indicated that there were no highly noticeable differences in the spontaneous use of the affected upper limb in relation to the less affected one on the main outcome measure (mini-AHA), indicating no significant functional difference between the hands; this was also a reason for exclusion. We also ensured that none of the recruited children were enrolled in other physical therapy programs during the study. These eligibility criteria were designed to ensure that any observed effects could be attributed with greater confidence to the hybrid therapy being evaluated, without confounding influences from other interventions.

The study was approved on 30 October 2020, by the Ethics Committee of the University of Malaga (Reference N: 75-2020-H), in accordance with the World Medical Association Declaration of Helsinki.

### 2.3. Early Infant Hybrid Therapy (eI-Hybrid)

The early intervention eI-Hybrid lasted 50 h for 10 weeks, with the participation of the family at home. The children would be motivated to perform the arranged activities in two periods of 30 min each day (from Monday to Friday). The first 30 min consisted of single-handed activities, following mCIMT considerations; these would be followed (not continued) by activities aimed at using both hands for 30 min, based on the considerations of BIT.

**Figure 1:**
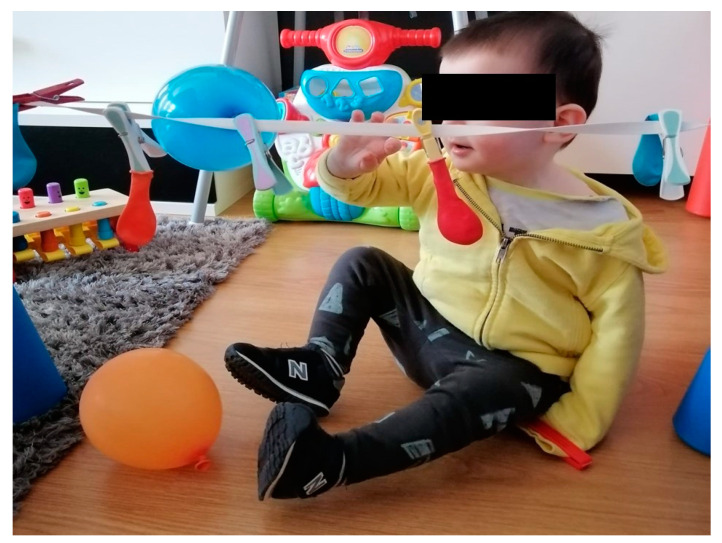
Long-sleeved T-shirt restriction in left upper limb for use of affected upper limb, inducing shoulder flexion into specific activity to touch balloons.

In the first part, the restriction in the less-affected upper limb was be achieved by using a long-sleeve T-shirt with a clip at the end of the sleeve (Figure 1). The restraint was only used during the session. The toys were first be placed close to the baby’s hand to facilitate contact, and then at a greater distance, when the child gained greater manual skill. To monitor improvement in manual skills, an approach based on the following three key indicators was used and explained to the families: the time it took the child to reach and manipulate the toys, the assistance provided by parents, and the need for compensatory postural adjustments.

On the other hand, in the BIT program, children with a lower ability will start with bimanual reaching and holding. The objects will need to be easy to grasp so that the children can hit, crash, or shake them. The toys will be selected based on the child’s age and motor ability. For those children with a lower level of motor skills, the toys will need to be presented within reaching distance to promote reaching and touching. In this case, the activities performed (Table 1) will be designed to train the desired components of movements required to complete the goal tasks and to be fun and motivating to facilitate cooperation and learning.

Activities and positioning during the play session: Depending on the actions present in the chosen objective, as well as the child’s age and ability, the pediatric physiotherapist, together with the family, determined which activities would be worked on each week, increasing the difficulty if a better execution was acquired. The aim was for movements to be self-generated, voluntary, and challenging yet attainable. During the play session, the infant would be seated with an upright and symmetrical trunk to enhance the movement of the paretic upper limb. This may have taken place in a highchair, on the floor, or in an adapted seating device. The parents would stand opposite the child and on the affected side to offer toys in different positions to increase reach distance and the range of upper limb movement. Time was provided for the infant to explore the objects and develop their own skills. To attract the child’s attention, toys with sound were be used.

### 2.4. Family Training

The home program is designed to align with motor learning principles, taking into the consideration of different factors within both the following interventions: the active involvement of the child and the family, and goal-oriented, structured, and individualized functional tasks suitable for the child, based on their interests, motivation, and manual ability. The physiotherapist joins the family for choosing toys and setting play at an appropriate ability level and amount of repetitions of tasks [31]. The tasks include grasping, and unimanual and bimanual exploration and manipulation of the toys, so they need to have different features to prompt different actions. Goals are established with the Goal Attainment Scale (GAS) [26,27] based on their daily routines. In addition, to train families to perform the therapy at home, training will be given beforehand, with a duration of two days, for 4 h each day, during which the therapy will be explained and the potential activities to be planned during eI-Hybrid are aligned with the child’s motivations and preferences, and will be taught. To make the task easier, and improve the family’s confidence, adjustments to the initial positioning of the child can be made, such as avoiding the inclination and anteriorization of the trunk, using an appropriate seat and the right height in relation to the table (for those activities that are carried out in sitting position). There can also be modifications to the object choice and their positioning will be close to the child at the start of the treatment. Prior to the start, each family will be asked about their child’s interests and preferences with regard to playing, which activities the child found the most difficult, and what motivates or frustrates them. On the day of training, they will create a list from those interests and preferences to show the parents how they should perform them during the intervention: what the more-affected arm/hand should do and whether it should assist or be the main hand during the bimanual task.

### 2.5. Supervision of Families

A pediatric physiotherapist will perform a weekly review through online sessions aimed at solving queries the parents could have about the different therapies (unimanual or bimanual activities, restraint type or difficulties in its use, massage strokes, and so on), changing toys to increase difficulty, or providing the child with other motor actions and enhancing the positioning of the child, all of this taking place through the reviewing of the videos of the proposed games.

Families could have time to comment on their experiences, difficulties, and how they feel during the implementation of the intervention. In addition, each family will have to fill out a monitoring sheet, including the toys used in each session and what objectives were set for their use, how the infant was positioned, how their interaction was (rejection, tired, active), the time spent on it (to check the adherence of the child and the family in the therapy), and compliance with the dosage.

Data collection was conducted through the monitoring sheets filled out by the families, which were collected at the end of each session. These sheets provided quantitative data on the toys used and the duration of activities, as well as qualitative insights into the family’s experiences and challenges.

### 2.6. Outcome Measures

#### 2.6.1. Feasibility

The feasibility of intervention measures are outlined as follows:

1. Acceptability: we evaluated the comprehensibility of the rules of the game regarding preparation and execution. This was measured using video analysis during the planning of the games, focusing on how to position the child on the table and their orientation to facilitate task execution.
2. Training compliance: The compliance with the intervention duration (10 weeks) was documented through weekly reviews. These included oral reports from parents regarding their adherence to the therapy schedule and confirmation of completed sessions.
3. Training smoothness: We examined the smoothness of activities by recording the number of problems and difficulties encountered. These data were be gathered through weekly documents that assessed task execution times and noted instances of rejection or interest in activities.
4. Training motivation: The motivation and perceived effort of the child was evaluated through direct observation during therapy sessions. Observers recorded behavioral indicators of engagement, and parents completed a questionnaire about their expectations prior to therapy and after the 50 h of intervention. This questionnaire contains five questions, each with five possible answers (see Supplementary Materials).

#### 2.6.2. Procedure Outcomes

The administration and evaluation procedure for the various tests used is described below. All outcome measures were administered and scored by trained, expert raters. In the case of the mini-AHA and AHA assessments, these are used to record a play session that was then be scored by a certified rater, unaware of the group assignment.

Outcome measures were be collected at 3 time points (week 0, week 10, and 6 months post-treatment), except for functional goals and participant satisfaction and expectations, which were be measured at week 0 and week 10.

##### Mini Assisting Hand Assessment (mini-AHA) Outcome

The Bimanual Functional Performance (BFP) was be assessed using the infant version of the Assisting Hand Assessment scale (mini-AHA) [28], and the Assisting Hand Assessment scale (AHA) [29,30]. Both measurements were used to examine the impact of the treatment on the bimanual performance of the affected upper limb. The mini-AHA is a validated, reliable, criterion-referenced test based on observation that measures how effectively infants with ages between 8 and 18 months with hemiplegia use their more-affected hand. The Assisting Hand Assessment v. 5.0 scale [32,33] was used in infants participating in therapy who were older than 18 months at the 6-month follow-up evaluation. To facilitate the interpretation of results, the logit scale is converted to a user-friendly 0–100 scale that is still Rasch-based and presents interval level data (namely, mini-AHA/AHA units). In addition, a clinically meaningful change is obtained with 5 AHA units of difference pre–post treatment scores.

##### Goal Attainment Scale (GAS)

The Goal Attainment Scale [26,27] was the tool used to measure functional goals. This is a widely used measurement tool to assess individualized attributes where no standardized measure exists. The GAS evaluates the degree to which goals have been achieved with the intervention.

Goal-setting procedure: prior to the intervention, a collaborative process was employed to establish specific functional goals for each child. This process involved several key steps:

1. Initial assessment: The therapeutic team conducted an initial assessment to understand the child’s current abilities and difficulties. This included gathering information from standardized assessments, clinical observations, and input from family members.
2. Family engagement: Families would actively participate in discussions about their child’s needs, preferences and aspirations. Through structured interviews or focus groups, family members expressed their priorities for therapy, focusing on functional activities that would enhance their child’s daily life.
3. Goal identification: Based on the information gathered, the therapy team and families would collaboratively identify specific, measurable, attainable, relevant, and time-bound (SMART) goals. Each goal was be tailored to the child’s individual circumstances and focused on improving functional independence in areas such as self-care, communication, and mobility.
4. Review and refinement: The identified goals were reviewed to ensure that they were realistic and fitted the child’s developmental stage. The therapy team provided feedback and guidance to help families refine their goals as needed.
5. Documentation: Each goal was documented, clearly stating the desired outcomes and how they would be measured. This documentation serves as a reference throughout the intervention to track progress and make adjustments as needed, as well as to propose new goals once achieved.

Each goal was scored from −2 (current level of performance) through 0 (desired outcome) to +2 (much greater than expected outcome). As part of the GAS goal-setting process, families were asked to state the degree of importance of individual goals to them, using the following 4 options: not at all important (score: 0), a little important (score: 1), moderately important (score: 2), and very important (score: 3). The therapists ranked the difficulty of the goals using the 4 following options: not at all difficult (score: 0), a little difficult (score: 1), moderately difficult (score: 2), and very difficult (score: 3). The therapists’ ranking of a goal’s difficulty was not communicated to the patient. GAS [34,35] is useful in evaluating pediatric therapy services and is responsive to change.

##### Satisfaction and Expectations from Parents 

Before performing the therapy, the parents had to complete a survey inspired by the Canadian Occupational Performance Measure (COPM) and referenced in the study by Ferre et al. [36]. This survey assessed their expectations regarding home-based therapies. After completing 50 h of therapy, parents filled out a second survey to evaluate their satisfaction with the program’s execution.

Both questionnaires consist of five questions, each of them with five possible answers depending on what question was asked (see Supplementary Materials).

### 2.7. Statistical Analysis

For the analysis of the feasibility outcomes, descriptive statistics were used to show general characteristics, with means (standard deviation) for quantitative data and percentages for qualitative data. A repeated-measures ANOVA test was performed on the BFP to observe the statistically significant difference between pairwise comparisons. The normality study was be undertaken through the Shapiro–Wilk test, and Greenhouse–Geisser adjustment was applied when the assumption of sphericity was violated (Mauchly’s test of sphericity, *p* < 0.05). Sphericity refers to the assumption that the variances of the differences between repeated measures are equal, which is required for accurate ANOVA results. When this assumption is violated, the Greenhouse–Geisser adjustment corrects for potential inaccuracies and reduces the risk of type I errors, ensuring a more reliable interpretation of the results.

The results are shown as the mean and standard deviation (SD), with a confidence interval of 95%. All analyses were performed by using the v.29 SPSS software package.

## 3. Results

**Figure 2:**
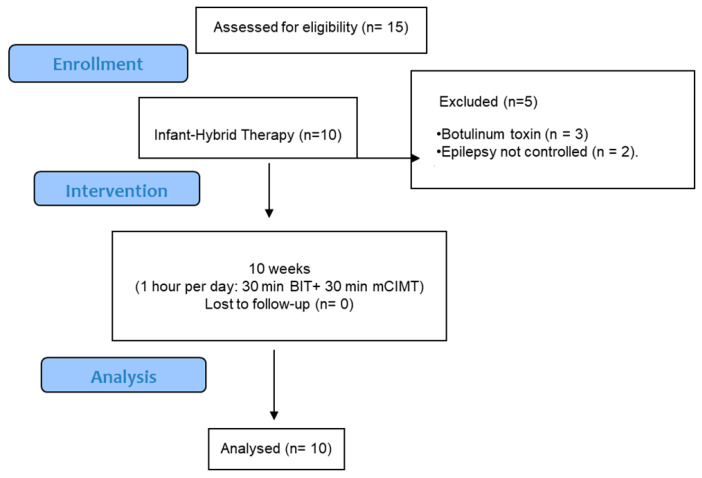
Infant hybrid flowchart.

Fifteen children were invited to participate in the study, but five were excluded. The reasons for exclusion were incidence of botulinum toxin (n = 3) and uncontrolled epilepsy (n = 2), as is shown in Figure 2. A total of 10 children with a mean age of 12.8 months (3.4) were enrolled and completed the study.

The age range of the parents was 34–45 years. Four families had completed higher education, and six families had a high school graduate or baccalaureate. The individual characteristics of the participants are shown in Table 2.

The age and mini-AHA basal situation scores are shown with the mean and standard deviation (SD) and interquartile range (IR). The rest of the results are shown in percentages (%). Extremely premature (37 gestational weeks); moderately premature (28–37 gestational weeks); not premature (>37 gestational weeks); perinatal stroke (from 20 weeks of fetal life to 28 days postnatal life); postnatal stroke (after 28 days postnatal life).

### 3.1. Feasibility Outcomes

Regarding the feasibility outcome, the following can be highlighted:

(1) Participation willingness: all eligible families (100%) agreed to join the project. (2) Participation rates/infants’ adherence (participants’ compliance with the dosed/prescribed exercise sessions in the program), and adverse event reports completed by parents: For the infant hybrid therapy, the rate of adherence was very high, at 97% (48.5/50 h), and there was no dropout during that period of treatment. Parents felt very satisfied with the intervention and no complication was added when they were asked about the stressful situations during the program. A dose of 90–95% was a high adherence, taking as a reference the study by Ferre et al. [37]. (3) Assessment procedures: All outcome measures were collected during the assessments and there were no missing data. (4) Assessment time scale: follow-up data were collected as planned. (5) Follow-up dropout: There was no follow-up dropout for any participant who finished the training. All parents understood and completed the weekly online review.

1. Acceptability: families reported adequate preparation and execution of activities, with no complications encountered.
2. Training compliance: all children successfully completed the therapy within the designated 10-week period.
3. Training smoothness: families demonstrated a clear understanding of how to implement activities with their infants. Initial suggestions were provided to enhance both children’s and parents’ positioning at the beginning of treatment, leading to smooth execution without complications.
4. Training expectations and functional goals: Parents’ expectations (Table 3), captured through questionnaires, evolved over time. Initially, many felt unprepared to carry out the therapy; however, as they gained self-confidence through initial training and ongoing weekly reviews with physiotherapists, their involvement in the intervention improved significantly.

All questions asked to parents obtained a significant value after 10 weeks of treatment, with the highest increase (2.4/5 points) observed in question 4 (Q4), related to the parent’s tolerance for the intervention.

### 3.2. Mini Assisting Hand Assessment (Mini-AHA) Outcomes

The bimanual functional performance score measured with the mini-AHA scale showed improvements after 10 weeks of treatment (*p* = <0.001), with an increase (9 units). The effect size was moderate (Cohen’s d = 0.53), showing significant intra-group changes in mini-AHA/AHA scales after intervention (week 10) and at the 6-month follow-up assessment compared to the baseline assessment (Table 4). Given that the overall range of the mini-AHA scale is 0 to 100 units, an increase of 9 units represents approximately 9% of the maximum possible score. This change can be significant in terms of assisting hand functionality, indicating an improvement in the child’s ability to perform bimanual tasks more effectively.

At the 6-month follow-up, the mean score was 50.3 (SD: 5.6), with a *p*-value of <0.001, showing that the gains in hand functionality were maintained over time (from basal situation to follow up at 6 months), suggesting the long-term positive effects of the intervention, because the decrease of 0.6 points from post-treatment to follow-up was not clinically relevant. Since the BFP measured with the mini-AHA/AHA scales in the following does not change significantly, upper limb function was maintained at a similar level.

### 3.3. Goal Attainment Scale (GAS)

Each family set a goal to achieve after intervention. The categories of the functional objectives proposed by the ten families belonged to leisure activities and home games (throwing a ball with both hands (two families), playing with sand in the park using both hands (three families), and building towers (three families)) and goals related to feeding (holding a glass with both hands (two families).

In Table 5, the different levels of goals (−2 to 2) set by families are defined.

Scores according to the difficulty, importance, and level of achievement are shown in Table 6 for each goal. All achievements were more than expected (score 1 or 2). Raw GAS scores at baseline and follow-up were transformed into GAS-T scores by using the established GAS methodology [34,35]. The transformation to a T score enables the comparison of performance across a population of patients who each have very distinct qualities and numbers of goals. The T value is a standardized score with a mean of 50 and a standard deviation of 10. Individual GAS-T scores range from <35 (much worse than expected) to >65 (much better than expected), with a score at or near 50 indicating that the goal was achieved as expected. All families scored −2 for each goal in the basal situation. Only for throwing a ball with both hands did two families achieve a score of 2, with a GAS T-Score of 70 points. The other goals achieved 60 points, with the four objectives being significant for families after infant hybrid therapy (Table 7).

## 4. Discussion

The objective of this study was to assess the bimanual functional performance increase in the affected upper limb and the feasibility of infant hybrid therapy applied at home with family involvement in children with UCP aged 9–18 months. The results demonstrated a significant improvement in mini-AHA scores, increasing from 41.9 at baseline to 50.9 post-intervention, and were at 50.3 at the 6-month follow-up (*p* < 0.001), indicating the long-term benefits of this family-centered intervention.

Also, it can be observed that there was great adherence in applying the intensive therapy, with participants achieving 97% of the total dose (48.5/50 h). Thus, the involved family is an important aspect to consider in intensive therapy, when focusing on family-centered programs. The high rate of adherence suggests that the active involvement of family members not only facilitates compliance with the protocol but may also enhance the effects of the treatment. This approach allows caregivers to make decisions and adjust therapy according to the times when the child is most attentive or active, thus optimizing the use of the affected limb in natural contexts. This is acknowledged in the satisfaction questionnaire in the question related to parents’ tolerance in therapy applications, where the best increase was obtained after treatment, since the families were very satisfied with the intensive therapy and their weekly review with a pediatric physiotherapist. This means that the correct interaction using the weekly review between the pediatric physiotherapist and families was an important factor in avoiding any complications and directing families and infants in the proposed activities according to the parents’ perceptions and interests. The results regarding family satisfaction with the expectations of the therapy, tolerance to the intervention, and the need to repeat it were very favorable. They could be associated with the restriction used to immobilize the less-affected arm and the fear of rejection by the child, as well as a reduction in tolerance and adherence by both the child and the parents. However, there was great adherence and achievement of the functional objectives, as well as great satisfaction and desire to repeat the intervention, which could be due to the existence of a modified therapy, in which the restriction was only applied during the activities and subsequently where the freedom of use of both hands was allowed [38]. In addition, the favorable results in bimanual functional performance and improved use would be an indication of wanting to repeat the intervention. This desire could also be due to the weekly review that the families had and the continued support that allowed their empowerment and security when carrying out the intervention and complying with it. This coaching process also occurs in the study by Svensson et al. [39], improving the understanding of the therapy and the family–therapist–child interaction to enhance compliance.

The family-centered approach is aimed at coaching and cooperation with caregivers, might increase treatment effects, and helps children to apply their learned skills to their natural context [40]. Moreover, when the family is participating in the treatment, they can make decisions and lead the treatment when the child is more attentive or active, encouraging their ability in their natural context to explore with the affected upper limb.

The establishment of functional objectives by the family in relation to the proposed therapy favors their involvement in the intervention to attain their goals, as well as in the satisfaction of attaining them, which is appreciated with the execution of infant hybrid therapy [41]. Setting objectives together with the family favors their adherence and satisfaction. Thus, the families highlighted the importance of being considered when establishing goals, and they are considered as facilitators. Experts and professional critical-care societies recommend sharing decision-making as the “best practice” based on the family’s goal of achieving patient value-congruent and well-informed decisions, and of reducing decisional conflict, decision regret, and potentially surrogate psychological distress. Setting goals according to the needs of families and making decisions based on this would increase adherence to training within the child’s natural context, since the family obtained a very high adherence rate, which could be due to setting goals proposed with eI-Hybrid and the weekly monitoring of the training program [40,41].

The restriction of the unaffected upper limb could be a factor to consider when seeking changes in bimanual functional performance in children with UCP at an early age. From a therapeutic perspective, this increase suggests a tangible improvement in the child’s ability to coordinate both hands in daily activities, which is crucial for the development of functional skills and independence. To provide a more complete understanding of how these improvements translate into specific benefits for daily living, it is important to consider how changes in BFP assessments correlate with improvements in concrete tasks such as eating, dressing, or playing. For example, an improvement in the BFP score may reflect a child’s increased ability to hold a utensil while reaching, manipulate objects during play, or button a piece of clothing. These skills are critical for autonomy and participation in daily activities, and the ability to perform bimanual activities more effectively can have a positive impact on a child’s quality of life and participation in daily activities.

The findings observed in the study of Eliasson et al. [42] showed children from 3 to 8 months old with asymmetric hand function and at high risk of developing UCP exhibiting a better development in the more-affected hand after 36 h of baby-CIMT compared to massage therapy; the treatment effect of baby-CIMT was moderate (Cohen’s d = 0.6). In our study, we observed similar trends, supporting the idea that intensive interventions can lead to sustained improvements in bimanual functional performance, even six months after treatment. Furthermore, it is noteworthy that children who received baby-CIMT were six times more likely to achieve a high functional level at two years of age compared to those in the massage group [43]. These results are consistent with previous studies mentioned which suggest that intensive therapies can yield lasting enhancements in hand functionality for children with UCP, underscoring the potential of approaches like baby-CIMT.

However, our contribution to the literature is significant for several reasons. Firstly, while the study by Eliasson et al. [42] focused on a population of children at high risk of UCP, our study focuses on children with confirmed UCP and applies a hybrid therapy modality combining mCIMT with intensive bimanual therapy (BIT). This hybrid approach has shown high adherence and effectiveness in the home context with active family involvement, which extends the applicability of the therapy to a more natural and less controlled environment. Furthermore, our study highlights the importance of family involvement in the implementation of the therapy. Although the study by Eliasson et al. [42] has demonstrated a positive effect of baby-CIMT, it did not address the impact of parental involvement on adherence and therapeutic outcomes. In contrast, our study shows that the active involvement of families may not only improve therapy adherence but also contribute to better functional outcomes in bimanual hand use in the child’s daily life. Thus, family-centered programs with specific training are associated with moderate improvements in both parenting and, particularly, child behavior in the short term, which could promote parent–child interaction and, therefore, greater interest in the execution of the therapy and its planning. Therefore, our findings contribute to the literature by demonstrating that the combination of an intensive home-based therapy with a family-centered approach may be equally effective or even more efficient than traditional clinical interventions, and they underline the importance of considering family involvement in the design of future therapies for children with UCP. Support for families of children with special needs refers to educational, social, and emotional support, in addition to support pertaining to health-related activities, knowledge and information, and children’s skill acquisition. It is parenting efficacy which contributes to increasing family empowerment, since this positive resource not only offers potential solutions for stressful situations but also improves the psychological confidence of the parent in their ability to effectively overcome said situations. Children with special needs receive the support of therapy from their families. In addition, the relationship between children with special needs and their families is mutually influential, meaning that the families are initially impacted by the children and the response of the families may affect the children in return. Thus, it is very important to understand the therapy procedure, its management, and how to carry it out [44,45]. For this reason, family empowerment can allow for an increase in functional performance and for the achievement of functional goals to be maintained in the long term, as the family acquires strategies through the learning obtained through coaching, enabling them to empower themselves and solve everyday problems, and increasing the spontaneous use of the affected upper limb in daily activities, as well as promoting the child’s participation within their natural context [46].

There are a few published studies on the application of combined therapies in infants [17]. It could be said that a 50 h dose would be sufficient to increase the changes in bimanual functional performance and maintain them in the long term, since they are stable at 6 months post-intervention in our study. While our study demonstrated that the 50 h dose of hybrid therapy led to stable improvements in bimanual functional performance at the 6-month follow-up, it is essential to consider that these outcomes may evolve over a longer period. Currently, there are no published studies on the long-term effects of combined therapies in infants. Future research should aim to monitor participants over extended periods to assess the sustained impact of hybrid therapy. Longer-term follow-up can provide insights into whether the benefits observed are maintained, increase, or decrease over time. Additionally, such studies could help determine the optimal duration and intensity of therapy needed for lasting improvements in bimanual functional performance. Investigating these aspects will be crucial for understanding the full potential of hybrid therapy and for developing more effective, evidence-based interventions for children with UCP. This concept could not be determined in the study by Chamudot et al. [47], in which an intervention of 56 h of mCIMT and BIT was carried out in children under 18 months of age and whose results for both treatments are equally effective in the functionality of the affected upper limb (without follow-up assessment). There were greater increases compared to our study in the mini-AHA scale, which could be a consequence of the fact that doses of 6 more hours were applied, or that the baseline situation of the children was much lower; these greater increases could also be due to the low bimanual functional performance (21–38 units) compared to the moderate bimanual performance observed in our study (39–62 units). This could mean that children with low bimanual functional performance obtain greater increases in mini-AHA after an intensive therapy program than children with moderate performance, which has not been studied in younger children, although other studies have addressed this in older children [48].

One of the limitations of the study was the single-group design and the small sample size, which raises concerns about the generalizability of the results to all populations with UCP. Due to the lack of a control group and the small sample, it is not possible to extrapolate the findings to a broader population. To address these limitations and improve generalizability in future studies, it is recommended to include comparison groups that receive only bimanual intensive therapy (BIT) and only constraint-induced movement therapy (mCIMT). This approach would allow for the evaluation of whether the combination of both therapies significantly enhances treatment effectiveness compared to the isolated application of each therapy. Additionally, recruiting a larger number of participants is suggested to increase statistical power and improve result representativeness, ensuring a diverse sample in terms of age and the severity of UCP to make the findings more applicable to a wider population. It is also important to consider other potential limitations, such as subjectivity in the assessment. While the primary outcome measures, including the mini-AHA, AHA, and GAS scales, were administered by trained therapists, ensuring an objective and standardized evaluation of outcomes, the assessment of adherence and satisfaction was reported by the parents through specific questionnaires. This reliance on parent-reported data introduces potential biases, such as social desirability bias, which can lead to an overestimation of the intervention’s efficacy and a more favorable presentation of the therapy experience than what truly occurred. Additionally, while families received ongoing support from a specialized pediatric physical therapist, the fact that the intervention was delivered by the parents could also influence the reported outcomes. Despite these limitations, a notable strength of the study was the application of uni- and bimanual activities within a home program with active parental involvement. This approach allowed for the exploration of the discovery of the use of the affected hand in its natural environment and subsequently improved bimanual performance fluency for activities of daily living. Future research should focus on conducting randomized controlled trials (RCTs) to compare hybrid therapy with BIT and mCIMT alone, explore different dosages of therapy to determine the most effective intervention schedule, and investigate long-term outcomes by following participants for 1–2 years post-intervention to assess the sustainability of motor function improvements. These steps will enhance our understanding of the optimal use of hybrid therapy and contribute to developing more effective early intervention protocols for UCP.

## 5. Conclusions

Infant hybrid therapy was found to be feasible for implementation at home with family involvement and satisfaction, leading to significant improvements in the affected upper limb in young children with UCP. This study indicates that a family-centered approach and the combination of therapies can be effective in enhancing bimanual functionality. The observed improvements in the Bimanual Functional Performance (BFP) score underline the importance of involving the family in the therapeutic process and tailoring interventions to maximize their impact.

The findings suggest that combining intensive bimanual therapy (BIT) with modified constraint-induced movement therapy (mCIMT) may offer additional benefits compared with using either therapy alone. Nevertheless, future research should examine the efficacy of the 50 h, 10-week regimen and explore whether adjustments in the dosage or combination of therapies could further improve outcomes, including using a larger sample of infants. This study provides a foundation for refining early intervention protocols for UCP to better meet individual and family needs.

## Figures and Tables

**Table 1:** Activities proposed to perform in infant hybrid therapy.

Movements	Unimanual Examples (Infant CIMT Part) Using More-Affected Upper Limb/Hand	Bimanual Examples (Infant BIT Part) Using Both Upper Limbs/Hands	Action Type
Shoulder flexion	Throw a ball, touch different objects located at different heights, touch balloons, reach different rattles, use toys that are offered at different distances.	Throw a big ball, touch different objects located at different heights, touch big balloons, put on necklaces, hats, reach different rattles, use toys that are offered at different distances.	Reaching actions
Elbow extension	Push a box with different weights, throw towers, put paint on paper at a distance so that it can stretch, throw a ball, reach different rattles, with toys that are offered at different distances.	Push a box with different weights, throw towers, throw a ball, reach different rattles, use toys that are offered at different distances.	Reaching actions
Forearm supination	Turn around a toy, put food in the mouth, give them objects in the palm of the hand, play the toy trumpet.	Move maracas or other toys between hands, manipulate an object, give them objects in the palm of both hands, put marbles in a bottle (held with the more-affected hand).	Manipulating actions and grasping actions
Wrist extension, finger extension, opening hand	Squeeze playdough, move sand, pick up sand and drop it, pick up a round rattle or ball, play the toy piano.	Squeeze playdough, move sand, pick up sand and drop it, pick up a round rattle or ball, play the toy piano, stretching plasticine, colliding two round toys, clapping while singing a song.	Reaching actions, manipuling actions, and grasping actions
Fine grasp	Move balls of different sizes, uncover objects by pulling the handle, pick up cereals, bits of biscuit.	Breaking pieces of paper, putting the marbles in the bottle with the more-affected hand, moving a small object from one hand to another hand.	Grasping actions

**Table 2:** Individual characteristics in children enrolled in infant hybrid therapy.

Early Infant Hybrid Therapy (N = 10)		
	Mean (SD)	IR
Age	12.8 (3.4)	9.8–16.3
Mini-AHA Basal situation	41–9 (7.7)	34.8–50.3
	N	%
Sex		
Female	6	60
Male	4	40
Affected upper limb		
Left	6	60
Right	4	40
Unilateral Cerebral Palsy cause		
Perinatal stroke	10	100
Postnatal stroke	0	0
Other	0	0
Birth (gestational age)		
Extremely premature	0	0
Moderately premature	3	30
Not premature	7	70
Associated disorders		
Epilepsy	0	0
Other disorders	0	0
No disorders	10	100

**Table 3:** Parents’ satisfaction outcomes.

Outcomes										
	Qo1		Qo2		Qo3		Qo4		Qo5	
	Pre	Post	Pre	Post	Pre	Post	Pre	Post	Pre	Post
Mean (SD)	3.4 (0.5)	1.6 (0.5)	4 (0)	2.0 (0)	4.4 (0.5)	1.4 (0.5)	3.5 (0.5)	1.1 (0.3)	3.0 (0)	1.0 (0)
*p* Value	0.004 *		0.002 *		0.002 *		0.004 *		0.002 *	

All values are expressed as the mean and standard deviation (SD). Qo, question; pre, pre-treatment; post: post-treatment; * indicates a value of statistical significance, when *p* < 0.05; BPF (0–100). Questions: Q1—how do you think the intervention will fit into your day to day? Q2—do you think your child will pay attention to the execution of the activities? Q3—how do you think your child will tolerate the intervention? Q4—how will you as a parent tolerate the intervention? Q5—do you think you will have the feeling of wanting to repeat it?

**Table 4:** Mini-AHA outcomes.

	Outcomes		
	Mini-AHA/AHA		
	Pre	Post	6 mo
Mean (SD)	41.9 (7.7)	50.9 (6.0)	50.3 (5.6)
*p* Value	<0.001 *		
Pre–Post	<0.001 *		
Pre-6 mo	<0.001 *		
Post-6 mo	0.072		

All values are expressed as the mean and standard deviation (SD). Pre: pre-treatment; post: post-treatment; 6 mo: 6-month follow-up after treatment. * indicates a value of statistical significance, when *p* < 0.05; mini-AHA/AHA (0–100).

**Table 5:** Levels of functional goals proposed by families.

Goal	+2	+1	0	−1	−2
Throwing a ball with both hands	Throws the ball with both hands	Shakes the ball with both hands	Raises the ball with both hands over the head	Touches the ball with both hands	Touches the ball with one hand
Playing with sand in the park using both hands	Picks up sand with both hands and moves it in the park	Drags sand with both hands in the park	Transfers sand from one hand to the other in the park	Touches the sand with both hands in the park	Touches the sand with one hand in the park
Building towers	Fits two Lego pieces together with both hands at home	Contacts the two Lego pieces with both hands at home	Puts the two Lego pieces together with both hands at home	Picks up a Lego piece with each hand at home	Picks up a Lego piece with one hand at home
Holding a glass with both hands	Brings the glass to the mouth with both hands	Lifts the glass with two hands	Drags the glass from the center of the table to the trunk with both hands	Touches the glass with both hands	Touches the glass with one hand

**Table 6:** Goal scoring according to the importance, the difficulty, the baseline situation, and the achievement after the intervention.

Goal	Importance	Difficulty	Baseline	Achieved	
Throwing a ball with both hands	0 1 2 3	0 1 2 3	−1: somewhat less than expected −2: much less than expected	Yes	Much better A little better As expected
				No	Partially achieved Same as baseline
					Worse
Playing with sand in the park using both hands	0 1 2 3	0 1 2 3	−1: somewhat less than expected −2: much less than expected	Yes	Much better A little better As expected
				No	Partially achieved Same as baseline
					Worse
Building towers	0 1 2 3	0 1 2 3	−1: somewhat less than expected −2: much less than expected	Yes	Much better A little better As expected
				No	Partially achieved Same as baseline
					Worse
Holding a glass with both hands	0 1 2 3	0 1 2 3	−1: somewhat less than expected −2: much less than expected	Yes	Much better A little better As expected
				No	Partially achieved Same as baseline
					Worse

Scoring is represented by blue color.

**Table 7:** GAS T-Score: baseline, achieved, and change in early infant hybrid therapy score.

Goal	Baseline GAS T-Score	Achieved GAS T-Score	Change in GAS T Score
Throwing a ball with both hands	30	70	40
Playing with sand in the park using both hands	30	60	30
Building towers	30	60	30
Holding a glass with both hands	30	60	30

Scores range from <35 (much worse than expected) to >65 (much better than expected), with a score at or near 50 indicating that the goal was achieved as expected.

## Data Availability

The original contributions presented in this study are included in the article/Supplementary Materials. Further inquiries can be directed to the corresponding author.

## Supplementary Materials

The following supporting information can be downloaded at https://www.mdpi.com/article/10.3390/jcm13226725/s1, Parent satisfaction questionnaire.

## References

[1] Odding E., Roebroeck M.E., Stam H.J. (2006). The epidemiology of cerebral palsy: Incidence, impairments and risk factors. Disabil. Rehabil..

[2] Johnson A. (2002). Prevalence and characteristics of children with cerebral palsy in Europe. Dev. Med. Child Neurol..

[3] Hoare B., Imms C., Randall M., Carey L. (2011). Linking cerebral palsy upper limb measures to the International Classification of Functioning, Disability and Health. J. Rehabil. Med..

[4] Nelson C.A., Sullivan E., Engelstad A. (2024). Annual Research Review: Early intervention viewed through the lens of developmental neuroscience. J. Child Psychol. Psychiatry.

[5] Houwink A., Aarts P.B., Geurts A.C., Steenbergen B. (2011). A neurocognitive perspective on developmental disregard in children with hemiplegic cerebral palsy. Res. Dev. Disabil..

[6] Akhbari Ziegler S., Hadders-Algra M. (2020). Coaching approaches in early intervention and paediatric rehabilitation. Dev. Med. Child Neurol..

[7] Palomo-Carrión R., Romay-Barrero H., Pinero-Pinto E., Romero-Galisteo R.P., López-Muñoz P., Martínez-Galán I. (2021). Early Inter-vention in Unilateral Cerebral Palsy: Let’s Listen to the Families! What Are Their Desires and Perspectives? A Preliminary Family-Researcher Co-Design Study. Children.

[8] Festante F., Antonelli C., Mazzotti S., Guzzetta A. (2023). Early Intervention in Cerebral Palsy: From Theory to Current Practice. Family-Centered Care in Childhood Disability: Theory, Research, Practice.

[9] Novak I., Morgan C., Adde L., Blackman J., Boyd R.N., Brunstrom-Hernandez J., Cioni G., Damiano D., Darrah J., Eliasson A.C. (2017). Early, Accurate Diagnosis and Early Intervention in Cerebral Palsy: Advances in Diagnosis and Treatment. JAMA Pediatr..

[10] Baker A., Niles N., Kysh L.M., Sargent B. (2022). Effect of Motor Intervention for Infants and Toddlers with Cerebral Palsy: A Systematic Review and Meta-analysis. Pediatr. Phys. Ther..

[11] Walker C., Shierk A., Roberts H. (2021). Constraint induced movement therapy in infants and toddlers with hemiplegic cerebral palsy: A scoping review. Occup. Ther. Health Care.

[12] Jackman M., Sakzewski L., Morgan C., Boyd R.N., Brennan S.E., Langdon K., Toovey R.A.M., Greaves S., Thorley M., Novak I. (2022). Interventions to improve physical function for children and young people with cerebral palsy: International clinical practice guideline. Dev. Med. Child Neurol..

[13] Charles J., Gordon A.M. (2006). Development of hand–arm bimanual intensive training (HABIT) for improving bimanual coordination in children with hemiplegic cerebral palsy. Dev. Med. Child Neurol..

[14] Araneda R., Ebner-Karestinos D., Paradis J., Klöcker A., Saussez G., Demas J., Bailly R., Bouvier S., de Tournai A.C., Herman E. (2024). Changes Induced by Early Hand-Arm Bimanual Intensive Therapy Including Lower Extremities in Young Children with Unilateral Cerebral Palsy: A Randomized Clinical Trial. JAMA Pediatr..

[15] Aarts P.B., Jongerius P.H., Geerdink Y.A., van Limbeek J., Geurts A.C. (2011). Modified Constraint-Induced Movement Therapy combined with Bimanual Training (mCIMT–BiT) in children with unilateral spastic cerebral palsy: How are improvements in arm-hand use established?. Res. Dev. Disabil..

[16] Palomo-Carrión R., Lirio-Romero C., Ferri-Morales A., Jovellar-Isiegas P., Cortés-Vega M.-D., Romay-Barrero H. (2021). Combined intensive therapies at home in spastic unilateral cerebral palsy with high bimanual functional performance. What do they offer? A comparative randomised clinical trial. Ther. Adv. Chronic Dis..

[17] Bansal A., Diwan S. (2021). Effect of Modified Constraint Induced Movement Therapy and Hand Arm Bimanual Intensive Training on Upper Extremity Skills and Functional Performance in Children with Spastic Hemiplegic Cerebral Palsy. Int. J. Health Sci. Res..

[18] Cohen-Holzer M., Katz-Leurer M., Meyer S., Green D., Parush S. (2017). The Effect of Bimanual Training with or Without Constraint on Hand Functions in Children with Unilateral Cerebral Palsy: A Non-Randomized Clinical Trial. Phys. Occup. Ther. Pediatr..

[19] Palomo-Carrión R., Romay-Barrero H., Lirio-Romero C., Arroyo-Fernádez R., M-Guijarro-Herraiz M., Ferri-Morales A. (2022). Feasibility of family-directed home-based bimanual intensive therapy combined with modified constraint induced movement therapy (h-BITmCI) in very low and low bimanual functional level: A brief report. Dev. Neurorehabilit..

[20] Afzal M.T., Amjad I., Ghous M. (2022). Comparison of classic constraint-induced movement therapy and its modified form on upper extremity motor functions and psychosocial impact in hemiplegic cerebral pals. J. Pak. Med Assoc..

[21] Nawge S., Karthikbabu S. (2023). Does bimanual task training benefit manual ability and hand function of children with bilateral spastic cerebral palsy?. J. Pediatr. Rehabil. Med..

[22] Steinbusch C.V.M., Defesche A., van der Leij B., Rameckers E.A.A., Knijnenburg A.C.S., Vermeulen J.R.J., Janssen-Potten Y.J.M. (2023). The Effect of Bimanual Intensive Functional Training on Somatosensory Hand Function in Children with Unilateral Spastic Cerebral Palsy: An Observational Study. J. Clin. Med..

[23] Dong V.A.-Q., Tung I.H.-H., Siu H.W.-Y., Fong K.N.-K. (2012). Studies comparing the efficacy of constraint-induced movement therapy and bimanual training in children with unilateral cerebral palsy: A systematic review. Dev. Neurorehabilit..

[24] Brauers L., Geijen M.M., Speth L.A., Rameckers E.A. (2017). Does intensive upper limb treatment modality Hybrid Constrained Induced Movement Therapy (H-CIMT) improve grip and pinch strength or fatigability of the affected hand?. J. Pediatr. Rehabil. Med..

[25] Sudati I.P., Sakzewski L., da Silva C.F.R., Jackman M., Haddon M., Pool D., Patel M., Boyd R.N., de Campos A.C. (2024). Efficacy and threshold dose of intensive training targeting mobility for children with cerebral palsy: A systematic review and meta-analysis. Dev. Med. Child Neurol..

[26] Kiresuk T.J., Smith A., Cardillo J.E. (1994). Goal Attainment Scaling: Applications, Theory, and Measurement. Lawrence Erlbaum Associ-Ates. https://www.routledge.com/Goal-Attainment-Scaling-Applications-Theory-and-Measurement/Kiresuk-Smith-Cardillo/p/book/9780898598896.

[27] King G.A., McDougall J., Tucker M.A., Gritzan J., Malloy-Miller T., Alambets P., Cunning D., Thomas K., Gregory K. (2009). An evaluation of functional, school-based therapy services for children with special needs. Phys. Occup. Therap. Pediatr..

[28] Greaves S., Imms C., Dodd K., Krumlinde-Sundholm L. (2013). Development of the Mini-Assisting Hand Assessment: Evidence for content and internal scale validity. Dev. Med. Child Neurol..

[29] Holmefur M.M., Krumlinde-Sundholm L. (2015). Psychometric properties of a revised version of the Assisting Hand Assessment (Kids-AHA 5.0). Dev. Med. Child Neurol..

[30] Krumlinde-Sundholm L., Eliasson A.-C. (2003). Development of the assisting hand assessment: A rasch-built measure intended for children with unilateral upper limb impairments. Scand. J. Occup. Ther..

[31] Schnackers M., Beckers L., Janssen-Potten Y., Aarts P., Rameckers E., van der Burg J., de Groot I., Smeets R., Geurts S., COAD Focus Group (2018). Home-based bimanual training based on motor learning principles in children with unilateral cerebral palsy and their parents (the COAD-study): Rationale and protocols. BMC Pediatr..

[32] Holmefur M., Aarts P., Hoare B., Krumlinde-Sundholm L. (2009). Test-retest and alternate forms reliability of the assisting hand as-sessment. J. Rehabil. Med..

[33] Krumlinde-Sundholm L., Holmefur M., Kottorp A., Eliasson A. (2007). The Assisting Hand Assessment: Current evidence of validity, reliability, and responsiveness to change. Dev. Med. Child Neurol..

[34] Churilov I., Brock K., Churilov J.M., Sutton E., Murphy D., MacIsaac R.J., Ekinci E.I. (2020). Goal attainment scaling outcomes in general inpatient rehabilitation: Association with functional independence and perceived goal importance and difficulty. J. Rehabil. Med..

[35] Harpster K., Sheehan A., Foster E.A., Leffler E., Schwab S.M., Angeli J.M. (2019). The methodological application of goal attainment scaling in pediatric rehabilitation research: A systematic review. Disabil. Rehabil..

[36] Ferre C.L., Brandão M., Surana B., Dew A.P., Moreau N.G., Gordon A.M. (2017). Caregiver-directed home-based intensive bimanual training in young children with unilateral spastic cerebral palsy: A randomized trial. Dev. Med. Child Neurol..

[37] Ferre C.L., Brandão M.B., Hung Y.-C., Carmel J.B., Gordon A.M. (2015). Feasibility of caregiver-directed home-based hand-arm bimanual intensive training: A brief report. Dev. Neurorehabilit..

[38] Reiss A.P., Wolf S.L., Hammel E.A., McLeod E.L., Williams E.A. (2012). Constraint-Induced Movement Therapy (CIMT): Current Perspectives and Future Directions. Stroke Res. Treat..

[39] Svensson K., Eliasson A.-C., Sundelin H., Holmqvist K.L. (2024). Parents in the Driver’s Seat—Experiences of Parent-Delivered Baby-mCIMT Coached Remotely. J. Clin. Med..

[40] Darrah J., Law M.C., Pollock N., Wilson B., Russell D.J., Walter S.D., Rosenbaum P., Galuppi B. (2011). Context therapy: A new intervention approach for children with cerebral palsy. Dev. Med. Child Neurol..

[41] Löwing K., Hamer E.G., Bexelius A., Carlberg E.B. (2011). Exploring the relationship of family goals and scores on standardized measures in children with cerebral palsy, using the ICF-CY. Dev. Neurorehabilit..

[42] Eliasson A.-C., Nordstrand L., Ek L., Lennartsson F., Sjöstrand L., Tedroff K., Krumlinde-Sundholm L. (2018). The effectiveness of Baby-CIMT in infants younger than 12 months with clinical signs of unilateral-cerebral palsy; an explorative study with randomized design. Res. Dev. Disabil..

[43] Nordstrand L., Holmefur M., Kits A., Eliasson A.-C. (2015). Improvements in bimanual hand function after baby-CIMT in two-year old children with unilateral cerebral palsy: A retrospective study. Res. Dev. Disabil..

[44] Mohammadzadeh E., Varzeshnejad M., Masoumpour A., Ahmadimehr F. (2023). The impact of the family-centered empowerment model on the children’s quality of life with chemical burns and their parent’s perceived stress. Burns.

[45] Ghazavi Z., Minooei M.S., Abdeyazdan Z., Gheissari A. (2014). Effect of family empowerment model on quality of life in children with chronic kidney diseases. Iran J. Nurs. Midwifery Res..

[46] Miller V., Sampson M.A., Howell D., Kitzman P. (2024). Coaching to Support Children with Disabilities in Occupational Therapy: A Literature Review. Occup. Ther. Health Care.

[47] Chamudot R., Parush S., Rigbi A., Horovitz R., Gross-Tsur V. (2018). Effectiveness of Modified Constraint-Induced Movement Therapy Compared with Bimanual Therapy Home Programs for Infants with Hemiplegia: A Randomized Controlled Trial. Am. J. Occup. Ther..

[48] Hoare B., Imms C., Carey L., Wasiak J. (2007). Constraint-induced movement therapy in the treatment of the upper limb in children with hemiplegic cerebral palsy: A Cochrane systematic review. Clin. Rehabil..

