# Supplementary material for: Feasibility of Home-Based Early Infant Hybrid Therapy in Children with Unilateral Cerebral Palsy
Source: J Clin Med. 2024 Nov 8;13(22):6725. doi: 10.3390/jcm13226725 (PMC11594390; doi:10.3390/jcm13226725)
Supplement: Supplementary file 1 [file jcm-13-06725-s001.zip › jcm-3197413-supplementary.pdf]

Questionnaire for parents (pre-intervention)

Below are 5 questions related to the perspectives you have as a family on the viability of this therapy for your child. It's very easy, you just have to give a score from 1-5, with 1 being the best and 5 being the worst; having this scoring a different meaning for each question, which is shown below. Any questions you can ask your therapist 😊

<u>Fill in the next data:</u>

- Name of child:
- Date of birth:
- Group of intervention:
- Other considerations:

Now…Answer the questions about your PERSPECTIVES on the intensive therapy your child will perform

✔ **Q1- How do you think the intervention will fit into your day to day?**

1- Very easy

2- Easy

3- Difficult

4- Very difficult

5- Impossible

✔ **Q2- Do you think your child will pay attention to the execution of the activities?**

1- Very attentive

2- Quite attentive

3- Attentive

4- Not very attentive

5- Not attentive

✔ **Q3- How do you think your child will tolerate the intervention?**

1- Very Good

2- Quite good

3- Good

4- Bad

5- Very bad

- ✔ **Q4- How will you as a parent tolerate the intervention?**

  1- Very Good

  2- Quite good

  3- good

  4- Bad

  5- Very bad

- ✔ **Q5- Do you think you will have the feeling of wanting to repeat it?**

  1- Yes

  2- Probably yes.

  3- I don't know.

  4- I think not

  5- No

Questionnaire for parents (post-intervention)

Below are 5 questions related to your satisfaction and experience in the application of intensive therapy which your child performed and how you saw his/her behavior. It's very easy, you just have to give a score from 1-5, with 1 being the best and 5 being the worst;aving this scoring a different meaning for each question, which is shown below. Any questions you can ask your therapist 😊

<u>Fill in the next data:</u>

- Name of child:
- Date of birth:
- Group of intervention:
- Other considerations:

Now…Answer the questions about your SATISFACTION AND EXPERIENCE with the intensive therapy your child performed.

✔ **Was it easy to fit the intervention into your day to day?**

1- Very easy

2- Easy

3- Difficult

4- Very difficult

5- Impossible

✔ **How attentive has the child been in the execution of the activities?**

1- Very attentive

2- Quite attentive

3- Attentive

4- Not very attentive

5- Not attentive

✔ **How has the child tolerated the intervention?**

1- Very Good

2- Quite good

3- good

4- Bad

5- Very bad

- ✔ **How have you tolerated the intervention as a parent?**

  1- Very Good

  2- Quite good

  3- Good

  4- Bad

  5- Very bad

- ✔ **Do you want to repeat the treatment?**

  1- Yes

  2- Probably yes.

  3- I don't know.

  4- I think not

  5- No
